# Closing Editorial: Advanced Polymeric Materials for Pharmaceutical Applications III

**DOI:** 10.3390/polym16213004

**Published:** 2024-10-26

**Authors:** Konstantinos N. Kontogiannopoulos, Panagiotis Barmpalexis

**Affiliations:** 1 Laboratory of Pharmaceutical Technology, Division of Pharmaceutical Technology, School of Pharmacy, Faculty of Health Sciences, Aristotle University of Thessaloniki, 54124 Thessaloniki, Greece; kkontogiannopoulos@gmail.com; 2 Natural Products Research Centre of Excellence-AUTH (NatPro-AUTH), Center for Interdisciplinary Research and Innovation (CIRI-AUTH), 57001 Thessaloniki, Greece

This Special Issue on “Advanced Polymeric Materials for Pharmaceutical Applications III” brings together innovative research that demonstrates the growing importance of polymeric materials in pharmaceutical sciences. This collection features 19 articles covering various topics, including nanocarriers for drug delivery, antimicrobial polymers, polymeric wound dressings, advanced dental materials, and next-generation devices. In this closing editorial, we provide an overview of recent developments, the knowledge gaps addressed by this Special Issue, and some promising directions for future research.

Polymeric materials have consistently shown versatility in advancing pharmaceutical applications, especially in controlled and targeted drug delivery systems. This Special Issue has highlighted several studies on nano- and micro-sized carriers for drug delivery, showing their role in improving therapeutic effectiveness and reducing side effects. For example, Ashar et al. developed Ibrutinib-loaded nanoliposomes optimized using response surface methodology [1], while Jin et al. presented a review on the role of multifunctional polymeric micelles for cancer therapy, demonstrating their potential to enhance bioavailability and precision in treatment [2]. The research on mucoadhesive nanocrystals for simvastatin by Bakhaidar et al. further highlights the usefulness of polymers in localized delivery systems, addressing challenges in bioavailability and patient compliance [3]. Chrysafi et al. developed poly(lactic acid) block copolymers for long-acting injectables of risperidone, which shows promise for improving the sustained release of antipsychotic medications, providing a more consistent therapeutic effect for patients [4].

Beyond drug delivery, this Special Issue explored polymeric formulations with antimicrobial and wound-healing properties. Bao et al. synthesized polylactic acid oligomers with broad-spectrum antimicrobial effects [5], while Campanholi et al. optimized a natural polymer-based medicine from *Copaifera reticulata* for skin wound care [6]. These contributions emphasize the role of polymers as carriers and active participants in therapy, particularly in applications where microbial resistance and wound management are critical. Liu et al. encapsulated Ligustrazine, a natural compound with antioxidant properties, in a liposome–hydrogel system for preventing skin photoaging [7]. This work highlights the potential of polymers in dermatological applications, specifically in promoting skin health and protecting against environmental stressors.

The Special Issue also highlighted polymers in medical devices and dental applications. Chiesa et al. fabricated graphene nanoplatelet-based textured fibrous fabrics for next-generation devices, demonstrating the potential of polymer composites in healthcare [8]. Also, reviews on this subject by Kostić et al. [9] and Liu et al. [10] demonstrated the use of acrylate polymers and polyetheretherketone (PEEK) in dental prostheses, respectively, showing advancements in biomaterials for dentistry, focusing on improving biocompatibility and mechanical properties. Vindokurov et al. further illustrated how heat treatment can influence the properties of PEEK fabricated via fused filament fabrication for biomedical applications, providing insights into material optimization [11]. Manakhov et al. studied the immobilization and release of platelet-rich plasma from modified nanofibers, offering insights into wound healing and regenerative medicine [12]. These advancements in dental and medical device applications are crucial for improving patient care, as they provide enhanced performance and durability, which are essential for long-term medical use. The use of advanced polymers in these fields underscores the importance of developing materials that meet clinical requirements and enhance the overall quality of life for patients.

A significant gap in polymeric research has been the need for improved stability and targeted delivery mechanisms. Studies like those by Bărăian et al. [13], which focused on imprinted polymeric drug reservoirs for localized glioblastoma treatment, and Chrysafi et al. [4], which developed poly(lactic acid) block copolymers for long-acting injectables, addressed these challenges by providing novel formulations aimed at enhancing stability and targeting capabilities. Ravikumar et al. worked on inhibiting carvedilol precipitation using polymeric precipitation inhibitors, further advancing our understanding of stability challenges in formulation science [14]. Koromili et al. tackled the solubility enhancement of luteolin, a poorly soluble compound, by preparing amorphous solid dispersions, showcasing how polymers can be used to improve drug solubility and bioavailability [15]. The ability to develop stable formulations that can effectively deliver drugs to specific sites in the body is a key area of research, as it directly impacts treatment efficacy. The findings presented in this Special Issue contribute significantly to addressing these challenges, offering new approaches that could be translated into clinical practice.

Another interesting development featured in this Special Issue is the synthesis of advanced polymeric systems for applications beyond traditional drug delivery. Quesada-Pérez et al. used coarse-grained simulations to study the release kinetics of drugs housed in flexible nanogels, providing new insights into the kinetic parameters governing drug release [16]. Cui et al. developed pH-sensitive polymeric frameworks for ibuprofen delivery, which can adjust their drug release behavior according to the surrounding pH, thus offering a responsive approach to drug delivery [17]. Widodo et al. presented pregelatinized sago starch as a new excipient for pharmaceutical tablets, showing how natural polymers can be adapted for use in drug formulations with potential benefits in tablet production [18].

Kim et al. fabricated nanostructured polycaprolactone (PCL) films using a thermal imprinting technique, evaluating their antibacterial properties for potential applications in wound dressings [19]. This study adds to the growing body of evidence supporting the use of polymeric materials in developing antibacterial surfaces and devices. Bakhaidar et al. demonstrated the use of thiolated mucoadhesive nanocrystals for local delivery of simvastatin, which could have implications for targeted treatments in oral health or localized disease management [3]. Additionally, Kostić et al. reviewed using acrylate polymers in dental applications, providing a comprehensive overview of their properties and their role in enhancing dental material performance [9].

Despite the progress made until now in the usage of advanced polymeric materials for pharmaceutical applications, there are still areas that need further research. One such area is the translation of lab-scale formulations into clinically relevant products. While many of the Special Issue studies presented significant advancements, scalability and regulatory aspects must be addressed to ensure these innovations reach the market. Furthermore, developing polymers that better mimic biological systems, such as biodegradable and bioresponsive polymers, remains an important direction for future research. The study by Cui et al. on pH-sensitive polymeric frameworks for ibuprofen delivery is a step in this direction [17]. Still, more work needs to be performed to achieve dynamic, responsive systems that adapt to complex biological environments. Additionally, integrating advanced characterization techniques to better understand the interactions between polymers and biological systems is essential for optimizing these materials for clinical use. By developing a deeper understanding of these interactions, researchers can design more effective polymers with fewer side effects.

In conclusion, this Special Issue has demonstrated the potential of advanced polymeric materials to transform pharmaceutical applications. The future of polymeric materials in pharmaceuticals is undoubtedly bright, while the collaboration between material and pharmaceutical technology scientists, biologists, clinicians, etc., will be crucial in bringing these innovations from the lab to the bedside, ultimately improving patient care and outcomes.

At this point, we would like to express our sincere gratitude to all the authors, reviewers, and editorial members who contributed to this Special Issue. Their efforts have made this compilation of cutting-edge research possible, and we are confident that it will serve as a valuable resource for researchers and practitioners in the field. We also encourage continued collaboration and exploration in this dynamic field, as the advancements made here are just the beginning of what is possible with polymeric materials in pharmaceutical applications.
